# 2S Albumin Storage Proteins: What Makes them Food Allergens?

**DOI:** 10.2174/1874091X00802010016

**Published:** 2008-02-06

**Authors:** F. Javier Moreno, Alfonso Clemente

**Affiliations:** 1 Instituto de Fermentaciones Industriales (CSIC), C/ Juan de la Cierva 3, 28006 Madrid, Spain; 2 Estación Experimental del Zaidín (CSIC), Profesor Albareda 1, 18008 Granada, Spain

**Keywords:** 2S albumins, food allergy, IgE-binding proteins, epitope mapping, disulphide bonds

## Abstract

2S albumin storage proteins are becoming of increasing interest in nutritional and clinical studies as they have been reported as major food allergens in seeds of many mono- and di-cotyledonous plants. This review describes the main biochemical, structural and functional properties of these proteins thought to play a role in determining their potential allergenicity. 2S albumins are considered to sensitize directly *via *the gastrointestinal tract (GIT). The high stability of their intrinsic protein structure, dominated by a well-conserved skeleton of cysteine residues, to the harsh conditions present in the GIT suggests that these proteins are able to cross the gut mucosal barrier to sensitize the mucosal immune system and/or elicit an allergic response. The flexible and solvent-exposed hypervariable region of these proteins is immunodominant and has the ability to bind IgE from allergic patients´ sera. Several linear IgE-binding epitopes of 2S albumins spanning this region have been described to play a major role in allergenicity; the role of conformational epitopes of these proteins in food allergy is far from being understood and need to be investigated. Finally, the interaction of these proteins with other components of the food matrix might influence the absorption rates of immunologically reactive 2S albumins but also in their immune response.

## 1 INTRODUCTION

2S albumins, defined on the basis of their sedimentation coefficient [1], are a major group of seed storage proteins widely distributed in both mono- and di-cotyledonous plants. As storage proteins, they are deposited in protein bodies of developing seeds and are utilized by the plant as a source of nutrients (amino acids and carbon skeletons) during subsequent germination and seedling growth. Recent findings have demonstrated that 2S albumins can also play a protective role in plants as defensive weapons against fungal attack [2]. In addition to their physiological role in plants, these small globular proteins are becoming of increasing interest in nutritional and clinical studies. The amino acid composition of 2S albumin proteins from many plant species has revealed their high content of sulphur-containing amino acids [1]. Typically, 2S albumins show high levels of cysteine residues, ranging from 6 to 13 mol %; in some cases, the content of methionine is relatively high, as occur with 2S proteins from Brazil nut (*Bertholletia excelsa*) reaching values of 17 mol %. To improve the nutritional quality of legume seeds, usually compromised by a relative deficiency of sulphur-containing amino acids, some attempts to transfer 2S albumin genes from different sources by genetic engineering were carried out. However, some disappointed results were obtained; when a 2S Brazil nut albumin gene was transferred into soybean, the recombinant 2S protein kept its intrinsic allergenicity [3, 4]. It was clearly shown that people who reacted to Brazil nut extracts on standard skin-prick tests had similar reactions in response to extracts of transgenic soybean that contained recombinant 2S Brazil nut albumins. Consequently, the transgenic soybean was considered unsafe for human and animal nutrition. These studies revealed that

In recent years, some members of this protein family have been described as major food allergens (Table **1**) demonstrating their ability to bind IgE from allergic patients´ sera [5, 6]. Allergenic proteins from yellow (*Sinapis alba*, Sin a 1) and oriental mustard (*Brassica juncea*, Bra j 1), Brazil nut (Ber e 1), castor bean (*Ricinus communis, *Ric c 1) or white sesame seeds (*Sesamum indicum, *Ses i 1) have been classified as 2S albumins.

Several attempts to categorize plant food allergens on the basis of their three-dimensional structure [7], biological function [8] or protein families [9, 10] have been carried out. In particular, 2S albumins are grouped in the prolamin superfamily; other allergenic proteins included in this superfamily are the non-specific lipid transfer proteins, the α-amylase/trypsin inhibitors and the prolamin storage proteins of cereals [9]. Typically, members of this superfamily are characterized by the presence of a well-conserved skeleton of eight cysteine residues [11] and a similar three-dimensional structure enriched in α-helices [12]. In addition to the global folding, other structural and biochemical properties are shared by this protein superfamily and could be involved in the intrinsic allergenicity of some of their members, including the 2S albumins [13]. This review deals with those features thought to play a potential role in promoting allergenicity within the 2S protein family.

## 2 STRUCTURAL FEATURES OF 2S ALBUMINS

### 2.1 Primary Structure

As occurs with other groups of storage proteins, the 2S albumins show a high level of polymorphism. These proteins are generally encoded by a multigene family leading to numerous isoforms subjected to post-translational modifica-

tions, mainly derived from proteolytic processing; these isoforms may show considerable differences in their structures and biological properties. Nevertheless, it is possible to define the protein structure of a “typical” 2S albumin. These proteins are synthesized as a single larger precursor polypeptide of Mr~18-21 kD, which is co-translationally transported into the lumen of the endoplasmic reticulum. After the formation of four intra-chain disulphide bonds, involving eight conserved cysteine residues, the folded protein is transported into the vacuole where is subsequently processed to a polypeptide of Mr ~12-14 kD and eventually to the large and small subunits of Mr~8-10 and 3-4 kD, respectively [14]. Owing to the conserved skeleton of cysteine residues, the small and large subunits remain associated by two inter-molecular disulphide bonds in the mature form; other two intra-chain disulphide bonds are present within the large subunit. Fig. (**1**) depicts the typical disulphide bond mapping of the 2S albumins. The conserved scaffold includes that the third and fourth cysteine residues are consecutive in the polypeptide chain (large subunit) and the fifth and sixth cysteine residues are separated by only one residue. The inter-chain disulphide bonds are those formed between cysteine residues 1-5 and 2-3 whereas the intra-chain bridges are formed by the cysteine residues 4-7 and 6-8. A range of variants differing from the “typical” 2S albumins in their structure or mode of biosynthesis has been reported. Such is the case of the major methionine-rich albumin, SFA8, from sunflower (*Helianthus annuus*) in which post-translational processing seems to be limited to the removal of the signal peptide and the pro-region with no further proteolytic cleavage of the polypeptide chain into large and small subunits [15]. SFA8 is the only 2S albumin isolated and characterized to date that is composed of a single polypeptide chain [16]. Variation in disulphide bond formation of conglutin δ from lupin (*Lupinus angustifolius*) has been also described, with an additional free cysteine residue being present; in the case of 2S albumins from peas (*Pisum sativum*), the subunits PA1a and PA1b do not appear to be associated by disulphide bridges, being readily separated by chromatography under non-denaturing conditions [17].

A characteristic post-translational modification of 2S albumin proteins is the clipping observed at the C-terminal side of both subunits. C-terminal microheterogeneity has been described in 2S albumins from different plant species, including Brazil nut [18, 19], rapeseed (*Brassica napus*) [20-23], castor bean [24] and sesame [25]. This heterogeneity could either be due to the presence of different precursors, a shift in the position of the cleavage site during the maturation process, or due to the presence of carboxypeptidases in the protein bodies of the seeds, as reported for the α-chains of pea seed isolectins [26]. However, the precise number and characteristics of the proteolytic enzymes involved in these post-translational modifications still remains unclear.

Despite the skeleton of cysteine residues of 2S albumins being highly conserved, a relatively low amino acid sequence homology, within and among plant species, can be observed. Figs. (**2** and **3**) illustrate the alignment of the primary structure and the generated dendrogram based on amino acid sequence similarity of major allergens of the 2S protein family. As expected, regions spanning the cysteine residues showed the highest amino acid sequence homology whereas the regions showing the lowest amino acid sequence homology corresponded to i) the C-terminal of the small subunit, ii) the NH_2_-terminal of the large subunit and iii) that contained between the sixth and seventh cysteine residues within the large subunit. Overall, the degree of sequence homology of 2S allergens is low, ranging from 14 to 40 %, and does not reflect phylogenetic relationships, with the exception of 2S albumins belonging to the Brassicaceae family (i.e., Bra j 1, BnIa, Bra n 1 and Sin a 1) (Table **2**). A high amino acid sequence polymorphism has been also found in 2S albumins belonging to the same specie, as occur with allergens from sesame (Ses i 1 and Ses i 2) or castor bean (Ric c 1 and Ric c 3) (Table **2**).

### 2.2 Secondary and Tertiary Structure

Fourier transform infrared (FT-IR) spectroscopic and/or circular dichroism (CD) studies have demonstrated that 2S albumins from many plant species, including those from yellow mustard [27], rapeseed [28-30], radish (*Raphanus sativus*) [31], sunflower [32-34], Brazil nut [19, 34], peanut (*Arachis hypogea*) [35, 36], soybean (*Glycine max*.) [37], sesame [25] and oriental mustard [38] are rich in α-helix contents (35-50 %). NMR spectroscopic studies have been carried out in order to determine the global folding of 2S albumins from rapeseed (BnIb), peanut (rAra h 6) and Brazil nut (rBer e 1) [39-41]. Further NMR structural studies have allowed the determination of the high-resolution three-dimensional structure in aqueous solution of the precursor form of the 2S albumin from rapeseed (rproBnIb) as well as the structure of 2S albumins from castor bean (rRic c 3) and sunflower (SFA-8) [42-44]. These studies revealed that all

2S albumins adopt a common and compact three-dimensional structural scaffold comprising a bundle of five α-helices displayed in different regions (helices Ia, Ib, II, III and IV) and a C-terminal loop folded in a right-handed superhelix stabilized by four conserved disulphide bonds. Connecting the α-helices III and IV, there is an exposed and relatively short segment known as “hypervariable region” which has been described to be the most important antigenic region of the 2S albumins. However, its variability in length and amino acid composition observed in 2S albumins from different plant species suggests that it does not play any role in determining the folded structure [42]. As example, Fig. (**4**) shows the three-dimensional structure of the recombinant castor bean 2S allergen Ric c 3 (RCSB PDB entry: 1PSY) [42].

The structural homology within the protein family is high and such similarities have recently been exploited in protein modelling studies. Using the 2S albumin structures from rapeseed and castor bean as templates, structural models for the 2S albumins from Brazil nut and English walnut, and those from pecan nut (*Carya illinoinensis*) and peanut have been reported [45, 46]. Strikingly, the global fold is shown to be similar to that of other sulphur rich proteins from the prolamin superfamily like the non-specific lipid transfer proteins from wheat (various species of the genus *Triticum*) [47] or the bifunctional α-amylase/trypsin inhibitor from ragi (*Eleusine coranaca*) [48]. The pattern of eight cysteines in specific order appears to be a structural scaffold of conserved helical regions that would form a network of disulphide bridges necessary for the maintenance of the tertiary structure [49].

### 2.3 Epitope Mapping

The identification and characterization of immunodominant regions containing IgE-binding epitopes are essential in understanding the interaction of food allergens and the immune system and, hence, crucial for the development of specific allergen immunotherapy. Early epitope mapping studies were carried out in major allergens of yellow (Sin a 1) and oriental mustard (Bra j 1); these studies provided the characterization of a commonimmunodominant IgE epitope (QGPHVISRIYQTAT) located in the hypervariable region [50, 51]. Later on, Robotham *et al*. [52] reported a peptide of twelve amino acids (QGL**RGEE**ME**E**MV), also located in the hypervariable region of Jug r 1, which showed the ability to bind strongly IgE from walnut-allergic patients´ sera. Mutational analysis demonstrated that **RGEE** and an additional **E** were necessary for maximum IgE recognition. Despite low sequence homology within the hypervariable region, the amino acid sequence RGGEMEE seems to be well conserved in 2S albumins from several plant species such as pecan nut, cashew nut**(*Anacardium occidentale*), Brazil nut or castor bean [46] (Fig. **5**). On the other hand, Ses i 2 is the only 2S allergen reported to date which does not contain any linear IgE-binding epitopes spanning the hypervariable region [53]. The epitope mapping of this allergen is characterized by several linear IgE-binding epitopes located between the helices Ia and Ib of the small subunit and at the helix II of the large chain (Fig. **5**). Epitope mapping studies performed on 2S allergens from peanut (Ara h 2) [54, 55], Brazil nut (Ber e 1) [56, 57] and cashew nut (Ana o 3) [58] have revealed that other regions, in addition to the hypervariable region, can bind strongly IgE from allergic patients´ sera. Accordingly, a wide number of major linear IgE-binding epitopes distributed throughout the small and large subunits were identified in these allergens (Fig. **5**). These findings are in agreement with the fact that allergens must be multivalent to elicit an allergic response to sensitized patients [59]. It is important to draw attention to the fact that most studies involving epitope characterization of 2S albumins are mainly based on mapping of linear epitopes (Table **1**). Unfortunately, largely unexplored in terms of allergen epitope characterization are those of conformational nature, comprised of amino acids distant in the proteins primary structure but adjacent once the protein folds. Given the high resistance of 2S albumins to harsh conditions (see sections 3 and 4), it is very plausible that conformational epitopes may exist as suggested for Sin a 1 or Jug r 1 allergens [50, 52]. A more detailed knowledge of conformational epitopes within the 2S family could provide us implicit structural information relating to the antigen itself and the mode of binding; such information could be also useful for diagnosis and epitope-specific immunotherapy.

To sum up, it can be concluded that the highly conserved and compact structure of 2S allergens plays a demanding role in reinforcing the ability of these proteins to reach intact the gut immune system. According to the low degree of amino acid sequence identity observed across plant species, the primary structure *per se* may not prefigure common regions of (linear) IgE recognition. Nevertheless, the unstructured, flexible and solvent-exposed hypervariable region seems to constitute the IgE immunodominant recognition site in these proteins. It has been suggested that the accessible surface area of this region could be more relevant in determining allergenicity rather than in establishing overall conformational similarity [43-44].

## 3 STABILITY OF 2S ALBUMINS TO THE GASTROINTESTINAL TRACT ENVIRONMENT

2S albumins are thought to sensitize *via *the gastrointestinal tract (GIT) suggesting that these proteins can resist and survive, at least to some extent, to the harsh conditions (acid pH, denaturing effects of surfactants and proteolytic activities of digestive enzymes) within the GIT. Subsequently, these proteins could be absorbed in immunologically active forms by the gut, facilitating the exposition of these allergens to the immune system in order to sensitize a naïve individual and/or elicit an allergic response in a sensitised individual. Under acidic conditions, 2S allergens from rapeseed [29, 30], Brazil nut [34, 45, 60], sunflower [34, 61], soybean [37] and sesame [25] are stable and retain their three-dimensional structure. Several studies have demonstrated the high resistance of 2S albumins against pepsin digestion in simulated gastric fluid [25, 34, 43, 60, 62-66]. Likewise, Ara h 2 gave rise to resistant large fragments upon treatment with pepsin that contained intact IgE-binding epitopes [67, 68]. Sen *et al*. [67] showed that disulphide bonds of Ara h 2 contribute significantly to its overall structure and stability and, if disrupted, as occur in the presence of a strong reducing agent, lead to its rapid degradation by digestive enzymes. The digestibility of 2S allergens from sesame (Ses i 1) and Brazil nut (Ber e 1) has been deeply investigated. An *in vitro* gastrointestinal digestion model system which incorporated a second digestion phase (following pepsin digestion) by using the digestive enzymes trypsin and chymotrypsin in order to mimic the passage of allergens into the gut have been investigated [25, 65]. Although a limited proteolysis of both 2S allergens was observed, large fragments which could still contain IgE-epitope regions remained after digestion. These results indicated that the characteristic conserved skeleton of cysteine residues and, particularly, the intra-chain disulphide bond pattern of the large subunit, can play a critical role in holding the core protein structure together, even after extensive proteolysis. Lehmann *et al.* [36] reported that peanut 2S allergens, Ara h 2 and Ara h 6, contained core structures highly resistant to trypsin and chymotrypsin digestion; the 2S allergen from oriental mustard, Bra j 1, was also hardly affected by the digestive enzymes trypsin and chymotrypsin [38].

Unlike digestibility studies, little information is available in relation to either *in vitro* or *in vivo* gastrointestinal absorption of 2S allergens. Recently, we have investigated the absorption rates of the major 2S allergens from Brazil nut, Ber e 1, and sesame, Ses i 1, across human intestinal epithelial Caco-2 cell monolayers following gastrointestinal digestion *in vitro* [69]. This study revealed that substantial amounts of both food allergens were transported across the Caco-2 cell monolayers, suggesting that these proteins are able to reach the mucosal immune system in intact form, so that they would be able to sensitize the mucosal immune system and/or elicit an allergic response.

As a result of association of proteins with cell membranes and other food ingredients (matrix effect), particularly lipids and/or polysaccharides, the susceptibility to proteolysis of food allergens may be altered [70]. Such associations may either occur naturally in the food matrix due to processing or in the GIT during the digestive process [13]. In this sense, 2S albumins have been clearly demonstrated to bind or associate with lipids [33, 71]. Burnett *et al*. [72] showed a range of food allergens, including 2S albumins from Brazil nut and sunflower, to adsorb to model stomach emulsions, providing a further means of resisting the pepsin digestion, whilst all allergens tested were desorbed with the addition of bile salts when the duodenal environment was mimicked. These authors suggested that the desorbed protein could be denatured and bound to surfactants, possibly associated with the mixed micelles present in the duodenum, further impairing the duodenal digestion. Additionally, the oriental mustard 2S allergen, Sin a 1, strongly interacted with acid phospholipid vesicles which could result in a protective mechanism against proteolytic digestion of the allergen but also in an increased cellular uptake as the extracellular leaflet of the intestinal brush border membranes has larger amounts of such phospholipids [73].

In addition to their intrinsic properties as food allergens, the food matrix may also contribute to their allergenic nature either by facilitating a protein to reach the sites of immune action through the gastrointestinal mucosa or by contributing to the activation of immune cells [74, 75]. Recent studies have demonstrated that purified allergens alone does not induce *per se *an IgE response in animal models, indicating that the food matrix should be taken into account when developing models for allergenic potential assessment. van Wijk *et al*. [76] showed that purified peanut allergens possess little intrinsic immune-stimulating capacity as compared to a whole peanut extract in mice. These differences were attributed to a selective activation of antigen-presenting cells, indicating that soluble peanut allergens require the adjuvanting capacity of certain compounds within the food matrix to induce sensitization. Similarly, Dearman *et al.* [77] have recently shown that endogenous Brazil nut lipids are required for the induction of optimal antibody responses to Ber e 1 in mice.

## 4 Stability of 2S albumins to thermal processing

The stability to food processing has been described as an important attribute in the assessment of the intrinsic allergenicity of 2S proteins [25, 43, 78, 79]. Besides their contribution to the resistance to proteolysis, disulphide bonds of 2S albumins are thought to play a key role in the stabilization to thermal treatment by reducing the conformational entropy of the proteins in their denaturated state [80-82]. The number and distribution of disulphide bridges seems to be a major contributor to such resistance, as occur with other sulphur-rich proteins like the anti-carcinogenic Bowman-Birk protease inhibitors [83-85]. The electrostatic interactions, in addition to the presence of disulfide linkages, seem to play an important role in stabilizing the molecular structure of 2S allergens [86]. CD measurements revealed that 2S albumins retained most of their secondary structure and, following heating at ~85-95 ºC, only partial and reversible loss of α-helical structures was detected [25, 34, 36-38, 43, 60, 61, 78, 79]. In addition, CD spectra acquired in the near-UV region indicated that the tertiary structure of 2S albumins may be also fairly resistant to heating below 100 ºC [38, 78]. Differential scanning calorimetry (DSC) studies indicated that some 2S allergens are more thermostable at neutral than acid pH. Thus, 2S allergens from rapeseed showed denaturation temperatures at 101 ºC and 88 ºC at pH 6 and 3, respectively [87]. Similarly, the denaturation temperature of the main Brazil nut allergen, Ber e 1, exceeded 110 ºC at neutral pH whereas a fully reversible thermal denaturation was observed at 82 ºC under acidic conditions [60]; 2S albumins from sunflower were denatured at 118 ºC and 112 ºC at pH 7 and 3, respectively [61]. Strikingly, the immunoreactivity of roasted Brazil nuts did not differ from raw nuts suggesting the presence of highly resistant epitopes from 2S Brazil allergens to severe heat treatment [88]. It is also possible that the presence of other food components may contribute to the thermostability of the allergenic activity of 2S albumins in heat-processed Brazil nuts as observed in other members of the prolamin superfamily such as the non-specific lipid transfer proteins [89]. Overall, these data unambiguously indicate that 2S albumins might be able to retain linear and potentially conformational epitopes following heat treatment up to 100 ºC at neutral pH and, hence, their ability to trigger an allergic reaction in a sensitised individual would be essentially unaltered.

## 5 CLINICAL RELEVANCE AND CROSS- REACTIVITY OF 2S ALLERGENS

2S albumins from a variety of plant foods including tree nuts, grain legumes, spices, oil seeds and cereals have been reported to bind IgE from allergic patients´ sera (Table **1**).In last decade, Brazil nut 2S albumins attracted special attention from researchers and consumers when the gene encoding the 2S protein was transferred into transgenic soybeans in order to increase their levels of sulphur-containing amino acids; patients with a history of Brazil nut allergy had positive reactions to extracts of these genetically modified crops on skin prick tests and IgE-binding assays [3]. Later on, studies corroborating the potential clinical relevance of 2S albumins from different plant sources such as Brazil nut [4], mustard [90], sesame [91], almond (*Prunus dulcis*) [92] or peanuts [36] were carried out. Recently, 2S albumins from other sources have been reported as emerging food allergens; such is the case of proteins from oilseed rape (*Brassica napus ssp. oleifera*, Bra n 1) and turnip rape (*Brassica rapa ssp. oleifera*, Bra r 1) [93]. It is also important to highlight the fact that not all the 2S albumins should be considered major allergens. In a recent study, none of the patients´ sera from 23 individuals allergic to soybean was found to have IgE specific against soybean 2S albumins suggesting that these proteins are not major allergens within the patient population analyzed [66].

Incidents of hypersensitivity reaction to 2S food allergens have been described with increasing frequency. Although symptoms such as mild laryngeal irritation, urticaria and asthma have been reported, it is important to stress the relative frequency of severe systemic symptoms, including angioedema and anaphylactic shocks [91]. Although the basis of such severity is unknown, some authors have suggested the high resistance of 2S proteins to both proteolytic attack and food processing as possible explanation for persistence of their allergenic potency and ability to induce systemic symptoms [36, 94]. Additionally, the severity of symptoms has been reported to be affected by other characteristics such as amount of allergen ingested and absorbed, the matrix effect, degree or type of immune response as well as target organ sensitivity [95]. However, little information is available to establish a causal relationship between protein structure and clinical symptoms.

**Fig. (3):**
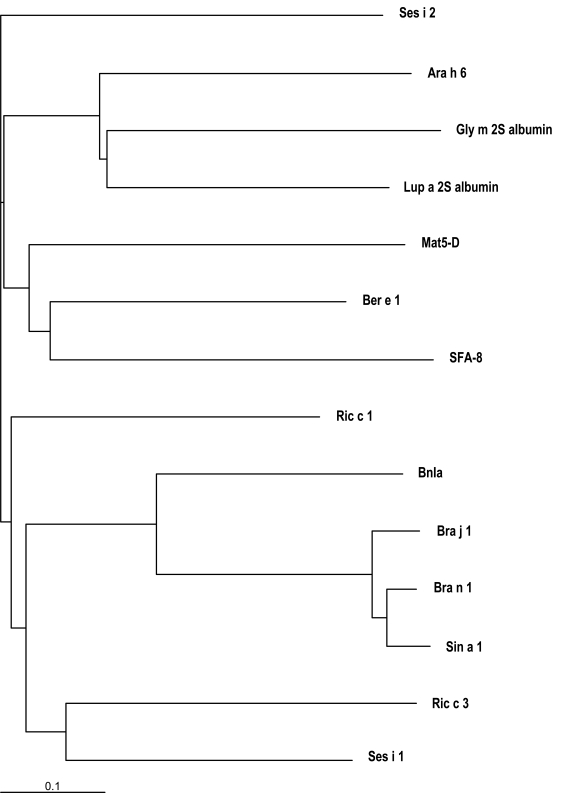
Dendrogram based on amino acid sequence similarity of allergenic 2S albumins from different species (Ara h 6, peanut; Ber e 1, Brazil nut; Bra j 1, oriental mustard; BnIa and Bra n 1, rapeseed; Gly m 2S albumin, soya; Mat5-D, upland cotton; SFA-8, sunflower seed; Lup a 2S albumin, lupin; Ric c 1 and Ric c 3, castor bean; Ses i 1 and Ses i 2, sesame seed; Sin a 1, yellow mustard) visualized by using TreeView [141]. The lower bar indicates 10% identity. Horizontal line distances between branch points reflect the degree of homology.

**Fig. (5):**
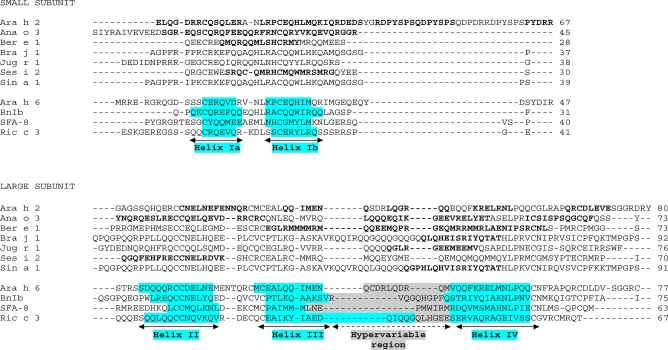
Alignment of the small and large subunits of allergenic 2S albumins whose linear IgE-binding epitopes (in bold) have been determined to date (Ara h 2, peanut; Ana o 3, cashew nut, Ber e 1, Brazil nut; Bra j 1, oriental mustard; Jug r 1, English walnut; Ses i 2, sesame seed; Sin a 1, yellow mustard). The α-helices Ia, Ib, II, III and IV (blue-shaded) and the hypervariable regions (grey-shaded) of Ara h 6 (peanut), BnIb (rapeseed), SFA-8 (sunflower) and Ric c 3 (castor bean) were taken from the 3D structure determined by NMR methods [40, 42-44]..

Although 2S albumins have high structural homology, cross-reactivity seems to be uncommon in this protein family (Table**1**). In a recent work, we obtained a polyclonal antisera against 2S albumins from Brazil nut and the ability to react against other nuts (almond, hazelnut, pecan, cashew, walnut and peanut) or legumes (pea and chickpea) was evaluated [88]; the cross-reactivity of antisera against all protein extracts tested was found to be negligible. These data support the fact that allergens with a similar fold are not necessarily cross-reactive [7]. Lack of cross-reactivity within this protein class has been attributed to the regions of sequential variability located mainly in the hypervariable loop and which are often the sites of IgE-binding [96, 97]. Aalberse [7] indicated that cross-reactivity between allergens is rare below 50 % amino acid identity and in most situations requires more than 70 % of sequence homology. Cross-reactivity between 2S allergens has been only demonstrated in cases of high sequence homology and seems to be linked to shared linear epitopes between allergens. For example, the 2S allergens from yellow mustard, Sin a 1, and rapeseed, Bra n 1, exhibit the highest sequence homology compared with other 2S proteins (Figs.**22-3**and Table**2**) and were recognized by IgE and IgG of sera of mustard-sensitive individuals as well as by immunoglobulins present in the serum of a rapeseed hypersensitive patient [98]. ELISA inhibition experiments clearly showed that these food allergens share common epitopes. In addition, cross-reactivity between 2S albumins of species from the *Brassicaceae* family, including oilseed rape, turnip rape and mustard, has been recently demonstrated [93]; the binding of a patients´ IgE to oilseed rape or turnip rape 2S albumin was effectively inhibited also by mustard 2S albumin. The peanut allergens Ara h 6 and Ara h 7, also belonging to the 2S albumin family, show 59 and 35 % amino acid sequence identity to Ara h 2, a well-known major food allergen, respectively [99]. EAST inhibition assays demonstrated significant cross-reactivity of Ara h 2 and Ara h 6 allergens [36]. Finally, a case of cross-reactivity between sunflower and mustard 2S albumins was reported in a serum from a patient allergic to mustard [100]. The same clinical group showed cross-reactive IgE binding to proteins with molecular mass of 10-12 kD between sesame and poppy protein extracts, suggesting that either Ses i 1 or Ses i 2 cross-reacts with a 2S albumin from poppy seed [101].

A joint FAO/WHO Expert Consultation on Allergenicity of Foods Derived from Biotechnology [102] proposed a decision tree based on a preliminary screening using computational sequence analysis to identify proteins with sequence similarity to known allergens. Thus, it was stated that cross-reactivity between a novel protein and a known allergen has to be considered when there is: i) more than 35% identity in the amino acid sequence of the mature protein (using a window of 80 amino acids and a suitable gap penalty); or ii) identity of at least 6 contiguous amino acids. According to these recommendations, only cross-reactivity among 2S *Brassicaceae* allergens would be expected within the 2S albumin family. However, it should be also necessary to consider that conformational epitopes distinct from continuous (linear) epitopes might also participate in cross-reactivity between 2S allergens.

## 6 FINAL REMARKS

The knowledge of common structural and physicochemical features of inherently allergenic protein families such as the 2S albumin could help to gain insight into the molecular basis of allergenicity, as well as to predict the potential allergenicity of novel and genetically modified foods. Extensive structural studies performed on 2S albumins have revealed that their compact and rigid structure dominated by a well-conserved skeleton of cysteine residues is responsible for their stability to the harsh conditions of the GIT. However, further epitope mapping studies, mainly focused on the characterization of conformational epitopes, are required to gain knowledge into their allergenic nature. Moreover, other environmental factors, such as the impact of food matrix or food processing on the 2S albumins structure, need to be investigated and correlated to their clinical response for better understanding of their route of exposure to the immune system.

## Figures and Tables

**Fig. (1):**
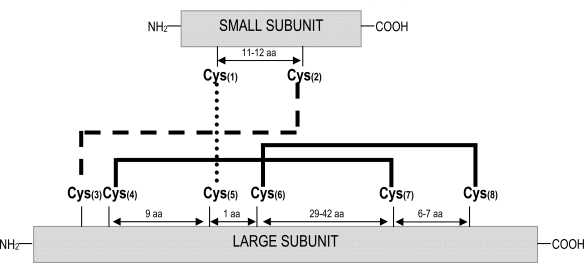
Schematic representation of the disulphide bond patterns formed between the eight conserved cysteine residues in the 2S albumin family.

**Fig. (2):**
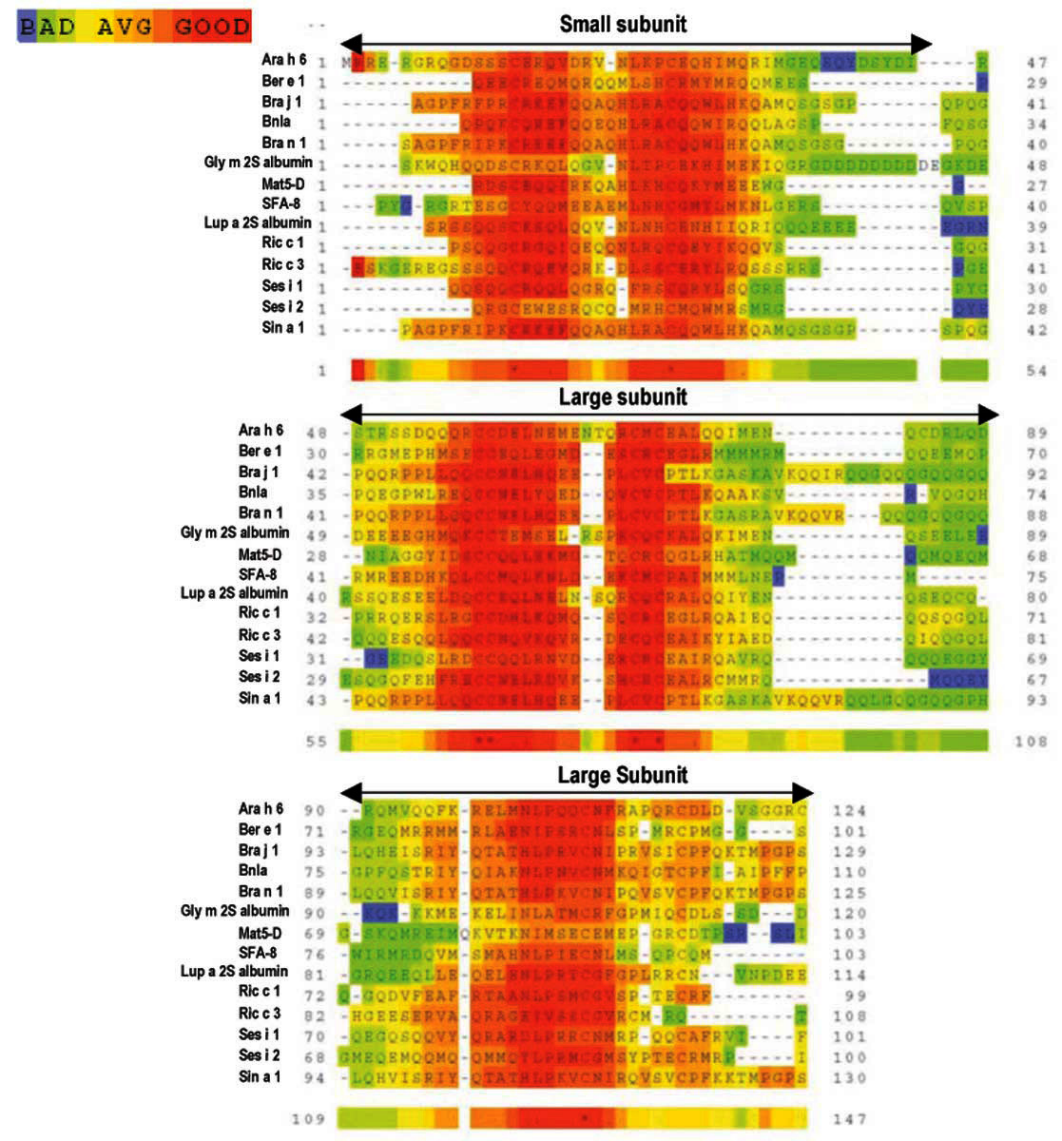
Multiple primary sequence alignment of allergenic 2S albumins from different species (Ara h 6, peanut; Ber e 1, Brazil nut; Bra j 1, oriental mustard; BnIa and Bra n 1, rapeseed; Gly m 2S albumin, soya; Mat5-D, upland cotton; SFA-8, sunflower seed; Lup a 2S albumin, lupin; Ric c 1 and Ric c 3, castor bean; Ses i 1 and Ses i 2, sesame seed; Sin a 1, yellow mustard) by using T-COFFEE (slow pair method) [140]. Numbers indicate the sequence position. Asterisks represent residue conservation among all the sequences.

**Fig. (4):**
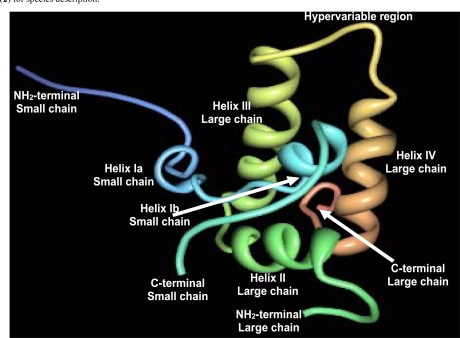
Schematic ribbon representation of the recombinant castor bean 2S allergen Ric c 3 (PDB entry: 1PSY) [ 42].

**Table 1:** Summary of Structural Properties of 2S Allergens

Allergen name	Species (common name)	Allergen designation	Epitope mapping	Cross-reactivity	Sequence accession number (ExPASy)	Mr of mature protein (kDa)	Subunits number	3D Structure Accession Number (RCSB/PDB)	Ref.
Ana o 3	*Anacardium occidentale* (Cashew nut)	Major	8 (linear)	Not known	Q8H2B8	12.6	2	Not determined	[58, 103]
Ara h 2	*Arachis hypogaea *(Peanut)	Major	10 (linear)	Other 2S albumins from peanut	Q6PSU2	17.3	Not determined	Not determined	[35, 36, 54, 104-107]
Ara h 6	*Arachis hypogaea*(Peanut)	Minor	Not known	Other 2S albumins from peanut	Q647G9	14.5	Not determined	1W2Q	[40, 99]
Ara h 7	*Arachis hypogaea*(Peanut)	Minor	Not known	Other 2S albumins from peanut	Q9SQH1 (Precursor)	15.8	Not determined	Not determined	[99]
Ber e 1	*Bertholletia excelsa* (Brazil nut)	Major	3 (T-cell epitopes) Helix III-hypervariable region-helix IV conformation is immunodominant	2S albumins from walnut, cottonseed, sunflower, castor bean	P04403	12.2	2	Not determined	[18, 19, 34, 45, 56, 57]
Bra j 1	*Brassica juncea* (Oriental mustard)	Major	1 (linear)	2S albumins from genus *Brassica*	P80207	14.6	2	Not determined	[51, 98, 108]
BnIa/BnIb	*Brassica napus* (Rapeseed)	Minor	Not known	2S albumins from genus *Brassica*	P24565	12.5	2	1PNB (global fold) 1SM7 (precursor form)	[21, 39, 43, 109]
Bra n 1 (BnIII)	*Brassica napus* (Rapeseed)	Major	Not known	2S albumins from genus *Brassica*	P80208	13.9	2	Not determined	[21, 30, 98, 110]
Bra n 2S albumin	*Brassica nigra* (Black mustard)	Major	Not known	Not known	Q42491 (Precursor)	Not determined	Not determined	Not determined	[111]
Bra r 1	*Brassica rapa* (Turnip)	Major	Not known	Not known	Q42473 (Precursor)	9.5-14.5	2	Not determined	[93, 111]
Car i 1	*Carya illinoinensis* (Pecan)		Not known	Not known	Q84XA9 (Precursor)	15.4	2	Not determined	[46, 112]
Cic a 2S Albumin	*Cicer arietinum* (Chickpea)	Major	Not known	Not known	Not determined	20.0	2	Not determined	[113, 114]
Fag e 10kD	*Fagopyrum esculentum* (Buckwheat)	Major	Not known	Not known	Q8W3Y9 (Precursor)	10.0	Not determined	Not determined	[115, 116]
Fag e 16kD	*Fagopyrum esculentum* (Buckwheat)	Major	Not known	Not known	Q2PS07 (Precursor)	14.6	Not determined	Not determined	[63, 117-119]
Gly m 2S albumin	*Glycine max* (Soya)	Minor	Not known	Not known	P19594	14.0	2	Not determined	[37, 66, 120-122]
CS-1A	*Gossypium herbaceum *(Arabian cotton)		Not known	Not known	Not determined	15.0	Not determined	Not determined	[123-124]
Mat5-D	*Gossypium hirsutum *(Upland cotton)		Not known	Not known	Q39787 (Precursor)	12.1	2	Not determined	[124-125]
SFA-8	*Helianthus annuus* (Sunflower seed)		Not known	2S albumins from mustard	P23110	12.1	1	1S6D	[16, 32, 44, 100, 126-128]
Jug n 1	*Juglans nigra* (Black walnut)		Not known	Not known	Q7Y1C2 (Precursor)	19.0	Not determined	Not determined	[129, 130]
Jug r 1	*Juglans regia* (English walnut)	Major	1 (linear)	Not known	P93198 (Partial sequence)	14.0	2	Not determined	[52, 131]
Pru du 2S Albumin	*Prunus dulcis* (Almond)	Major	Not known	Not known	P82944 (Partial sequence)	12.0	Not determined	Not determined	[92]
Ric c 1	*Ricinus communis* (Castor bean)	Major	Not known	Not known	P01089	11.3	2	Not determined	[132-134]
Ric c 3	*Ricinus communis* (Castor bean)	Major	Not known	Not known	P01089	12.0	2	1PSY	[24, 42, 133, 134]
Ses i 1	*Sesamum indicum* (Sesame)	Major	Not known	2S albumin from poppy seed	Q9AUD1	12.1	2	Not determined	[25, 101, 135]
Ses i 2	*Sesamum indicum* (Sesame)	Major	9 (linear)	2S albumin from poppy seed	Q9XHP1	12.5	2	Not determined	[53, 101, 135-137]
Sin a 1	*Sinapis alba* (Yellow mustard)	Major	1 (linear)	2S albumins from genus *Brassica*	P15322	14.2	2	Not determined	[50, 98, 138, 139]

**Table 2:** Number of Amino Acid Residues Conserved in 2S Albumin Allergen Sequences of Different Sources. The Maximum Number of Identical Contiguous Amino Acids is Shown in Brackets

Allergen^*^ name	Ara h 6	Ber e 1	Bra j 1	BnIa	Bra n 1	Gly m 2S albumin	Mat5-D	SFA-8	Lup a 2S albumin	Ric c 1	Ric c 3	Ses i 1	Ses i 2	Sin a 1
Ara h 6		27 (2)	24 (2)	23 (3)	24 (2)	45 (5)	25 (3)	28 (3)	50 (5)	32 (3)	25 (3)	31 (3)	28 (4)	22 (2)
Ber e 1	27 (2)		28 (2)	27 (3)	28 (2)	26 (2)	38 (3)	35 (3)	31 (5)	40 (7)	29 (2)	36 (4)	34 (4)	28 (2)
Bra j 1	24 (2)	28 (2)		58 (9)	115 (32)	17 (3)	23 (2)	25 (2)	23 (3)	39 (3)	33 (6)	37 (3)	31 (5)	115 (40)
BnIa	23 (3)	27 (3)	58 (9)		59 (9)	19 (2)	27 (2)	19 (3)	25 (3)	33 (3)	28 (4)	34 (3)	27 (5)	61 (9)
Bran 1	24 (2)	28 (2)	115 (32)	59 (9)		16 (3)	25 (2)	23 (2)	28 (3)	38 (3)	32 (6)	34 (2)	29 (5)	114 (37)
Gly m 2S albumin	45 (5)	26 (2)	17 (3)	19 (2)	16 (3)		26 (3)	21 (2)	48 (5)	27 (2)	27 (3)	24 (4)	23 (2)	18 (3)
Mat5-D	25 (3)	38 (3)	23 (2)	27 (2)	25 (2)	26 (3)		24 (2)	30 (3)	31 (4)	23 (2)	29 (5)	28 (3)	24 (2)
SFA-8	28 (3)	35 (3)	25 (2)	19 (3)	23 (2)	21 (2)	24 (2)		24 (4)	23 (3)	22 (2)	29 (2)	25 (2)	27 (2)
Lup a 2S albumin	50 (5)	31 (5)	23 (3)	25 (3)	28 (3)	48 (5)	30 (3)	24 (4)		35 (3)	33 (3)	28 (3)	29 (3)	23 (3)
Ric c 1	32 (3)	40 (7)	39 (3)	33 (3)	38 (3)	27 (2)	31 (4)	23 (3)	35 (3)		31 (4)	38 (4)	32 (5)	34 (3)
Ric c 3	25 (3)	29 (2)	33 (6)	28 (4)	32 (6)	27 (3)	23 (2)	22 (2)	33 (3)	31 (4)		39 (6)	23 (3)	33 (6)
Ses i 1	31 (3)	36 (4)	37 (3)	34 (3)	34 (2)	24 (4)	29 (5)	29 (2)	28 (3)	38 (4)	39 (6)		39 (5)	35 (2)
Ses i 2	28 (4)	34 (4)	31 (5)	27 (5)	29 (5)	23 (2)	28 (3)	25 (2)	29 (3)	32 (5)	23 (3)	39 (5)		29 (5)
Sin a 1	22 (2)	28 (2)	115 (40)	61 (9)	114 (37)	18 (3)	24 (2)	27 (2)	23 (3)	34 (3)	33 (6)	35 (2)	29 (5)	

*See legend of Fig. ( 2) for species description.
